# Tiny Yeast Comet 1-Dependent Polymers Suppress Taxol-Stabilized Microtubule Depolymerization Induced by Ice-Cold CaCl_2_

**DOI:** 10.3390/ijms27125436

**Published:** 2026-06-16

**Authors:** Scott C. Schuyler, Hsin-Yu Chen, Cheng-Ye Weng

**Affiliations:** 1 Department of Biomedical Sciences, College of Medicine, Chang Gung University, Kwei-Shan, Taoyuan 333, Taiwan; 2 Department of Otolaryngology—Head and Neck Surgery, Chang Gung Memorial Hospital, Kwei-Shan, Taoyuan 333, Taiwan; 3 Graduate Institute of Biomedical Sciences, Chang Gung University, Kwei-Shan, Taoyuan 333, Taiwan

**Keywords:** Tiny Yeast Comet 1 (Tyc1), p31^comet^, microtubule, mitotic-arrest deficient 2 (Mad2), cell division cycle 20 (Cdc20), Taxol, electron microscopy (EM), *Saccharomyces cerevisiae*

## Abstract

Budding yeast Tiny Yeast Comet 1 (Tyc1) was previously identified based on homology to human p31^comet^. The conserved homologous region is within the previously mapped p31^comet^ Taxol-stabilized microtubule binding domain at the C-terminus of p31^comet^. The p31^comet^ protein mimics the 3-dimensional shape of the HORMA-domain protein Mitotic Arrest-Deficient 2 (Mad2). Several HORMA-domain proteins have been reported to form polymers. By employing negative staining electron microscopy, we observed that the Tyc1 protein forms comet-tail-shaped polymers in association with Taxol-stabilized microtubules. When associated with microtubules, Tyc1p polymers frequently displayed a braid-like appearance around and off the ends of the microtubules. Analysis of two mutant forms of Tyc1p revealed that the amino acid motifs that are conserved between Tyc1p and human p31^comet^ were essential for robust polymer formation. Tyc1p polymers that were formed in the presence of Mad2p in association with a Mad2-binding motif peptide were able to suppress Taxol-stabilized microtubule depolymerization that was induced by exposure to ice-cold CaCl_2_. In conclusion, yeast Tyc1p forms polymers that can suppress Taxol-stabilized microtubule depolymerization, potentially yielding an insight into a microtubule-associated function for human p31^comet^.

## 1. Introduction

Tyc1p was identified based on homology to human p31^comet^ and is a non-essential gene in *Saccharomyces cerevisiae* that affects Anaphase-Promoting Complex/Cyclosome-Cell Division Cycle 20 (APC/C-Cdc20p) activity [1]. When Tyc1p was overexpressed in yeast it caused an increased sensitivity to the microtubule poison benomyl, and this sensitivity was enhanced in a *mad2Δ* mutant. In high-throughput screens and within systematic functional analyses, Tyc1p displayed evidence for physical interactions in vivo with Mad2p and with ABerrant Microtubules 1 (Abm1), among other proteins [2]. Tyc1p was also assigned to a BInding to Microtubules/End-Binding 1 (Bim1/EB1) functional group, which also includes Mad2p and Abmp1, with a moderate level of confidence [3]. Furthermore, a *tyc1* mutant allele has displayed genetic interactions with the *cbf2-2* allele, a known core kinetochore component [4]. These measured and modeled physical, functional, and genetic interactions from systematic analyses place Tyc1p within a nexus between mitotic microtubule plus-ends and kinetochore function. Furthermore, the spindle assembly checkpoint protein Mad2p, also well-established within this nexus, is localized to unattached kinetochores and promotes the formation of the Mitotic Checkpoint Complex (MCC) by forming a ‘safety-belt’ binding loop around the APC/C co-factor Cdc20p [5]. Although the in vivo phenotypes of *TYC1*, and the reported high-throughput physical/functional/genetic interactions with the *TYC1*/Tyc1 gene/protein are intriguing, no previous biochemical analysis physically connecting Tyc1p to microtubules has been reported.

By contrast, human p31^comet^ (originally named Caught by MAD2 Protein 2 (CMT2), but also referred to as p31(comet), MAD2L1BP, or Comet) has been observed to have a role in mitotic cell cycle regulation, has been demonstrated to associate with a Dynein Intermediate Chain DNCI2c, and with microtubules in vivo and in vitro [6,7]. In addition, p31^comet^ has been identified as a potential anti-cancer therapeutic target and mutations in p31^comet^ have been associated with cancer development [6]. The primary focus of research on p31^comet^ has been connected to MAD2-CDC20 mitotic cell cycle regulation. Working together with the TRIP13 AAA+ ATPase, p31^comet^ participates in the inactivation of the MCC, promoting the release of CDC20 from the MAD2 ‘safety-belt’ loop and thereby promoting entry into anaphase [8]. p31^comet^ also localizes to unattached kinetochores in a MAD2-dependent manner, and to the plus-ends of microtubules of the mitotic spindle where it co-localizes with EB1, the homologue of budding yeast Bim1p, a microtubule plus-end tracking protein (+TIPs) [7]. The p31^comet^ protein was originally stated to form “comet shaped” structures in cells, which hints that it may have a polymeric form [9,10,11]. These comet-shaped structures were observed extending several micrometers out from unattached mitotic kinetochores, and the presence of these structures required MAD2. In addition, there are several reports of p31^comet^ co-localizing with microtubules of the mitotic spindle, notably at the plus-ends where the GTP-cap is mimicked by Taxol-stabilized microtubules [7,9,10]. The p31^comet^ protein was also observed to bind to Taxol-stabilized microtubules in vitro, and the domain responsible for this activity resides in the C-terminal region of the protein, a region that contains two amino acid motifs (EDWFRPKLNYRVPSRGHKLTVTLS) sharing homology with Tyc1p (DDWFSRKFSKAVHGNNHGTISLS) [1,7]. Localization with the plus-ends of microtubules was reported to be enhanced by an association with a Dynein Intermediate Chain DNCI2c [7]. Notably, Dynein Intermediate Chains have been localized to the outer fibrous corona of kinetochores [12,13]. Finally, p31^comet^ mimics the structure of MAD2, and MAD2 is a HORMA-domain protein [11]. Some HORMA-domain proteins have been established to form polymers [13].

Although Tyc1p shares homology with p31^comet^, a biochemical analysis of Tyc1p focused on microtubule function has not been performed. While exploring the biochemical activity of Tyc1p in vitro, we observed by negative staining electron microscopy (EM) that Tyc1p forms “comet-shaped” polymers in association with microtubules that in the presence of Mad2p + a Mad2-binding motif peptide derived from Cdc20p called “DQ36” can suppress Taxol-stabilized microtubule depolymerization induced by ice-cold CaCl_2_ [1].

## 2. Results

### 2.1. Tyc1p Forms Extended Comet-Shaped Polymers in the Presence of Taxol-Stabilized Microtubules

**Figure 1:**
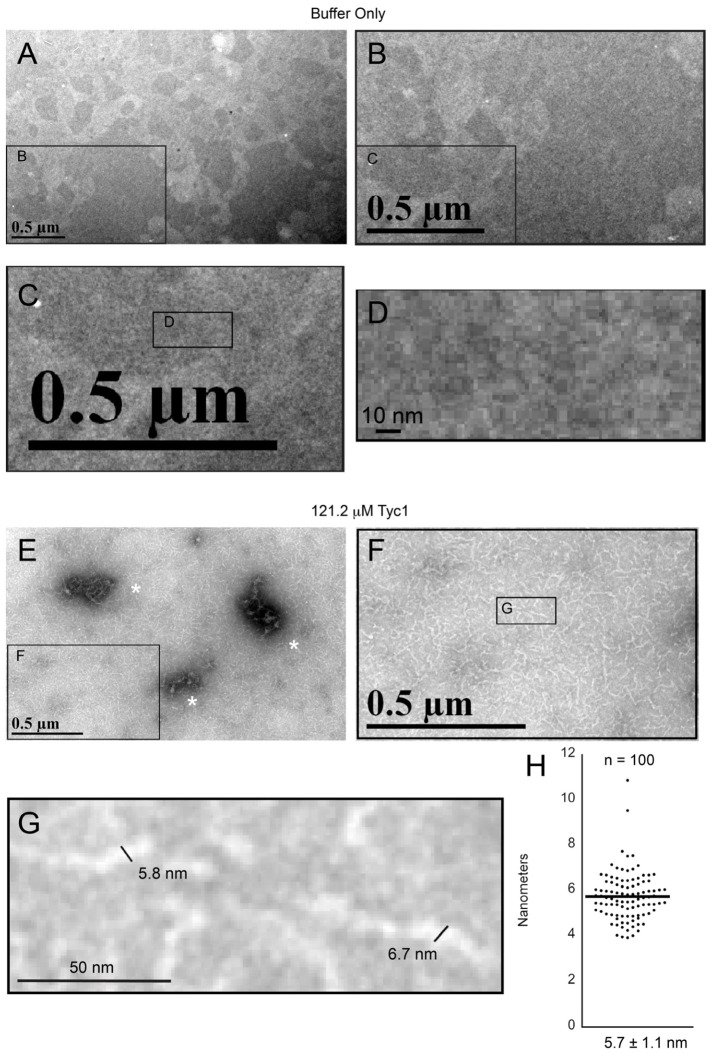
Tyc1p polymers have a diameter of 5.7 nm ± 1.1 nm (mean ± standard deviation). (**A**–**D**) The buffer-only control shown at larger and larger magnifications. No polymers were visible. (**E**,**F**) An example micrograph showing filamentous Tyc1p polymers of at 121.1 μM. A series of enlargements of the insets to display the polymer diameter. Asterisks “*” denote large aggregate structures. (**G**) Examples of measurements of the diameter of the polymers showing two examples, one with a measured diameter of 5.8 nm and a second with a diameter of 6.7 nm. (**H**) Quantification of the diameter of the polymers in (**G**).

To investigate if Tyc1p may be able to form polymers as other HORMA-domain proteins do, we employed negative staining electron microscopy to investigate and visualize pure Tyc1 protein directly at a high molar concentration (Figure 1). Linear polymers were visible by negative staining at 121 μM, as were dark clustered aggregates (marked with an *).

**Figure 2:**
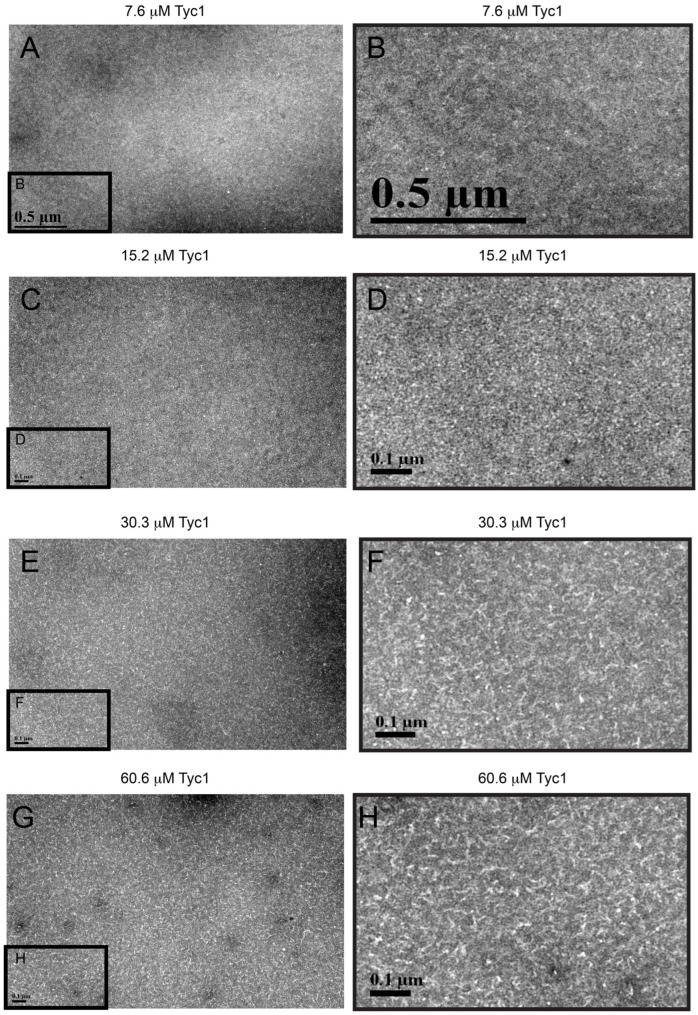
Tyc1p forms polymers at a concentration of about 30 μM. Increasing concentrations of Tyc1 protein were observed by negative staining EM. Scale-bars are displayed in the lower left corners. (**A**,**B**) Tyc1p at 7.6 μM. No polymers were evident. (**C**,**D**) Tyc1p at 15.2 μM. No polymers were evident. (**E**,**F**) Tyc1p at 30.3 μM. Tyc1p polymer formation became visible. (**G**,**H**) Tyc1p at 60.6 μM. Tyc1p polymers were evident. The scale bars in all images are 0.1 μm.

The observed Tyc1p polymers were strands scattered on the EM grid surface with a measured polymer diameter of 5.7 ± 1.1 nm (n = 100; mean ± standard deviation). We further investigated the minimal concentration needed to observe Tyc1p polymer formation by negative staining EM (Figure 2). The Tyc1p polymers became evident at about 30 μM.

**Figure 3:**
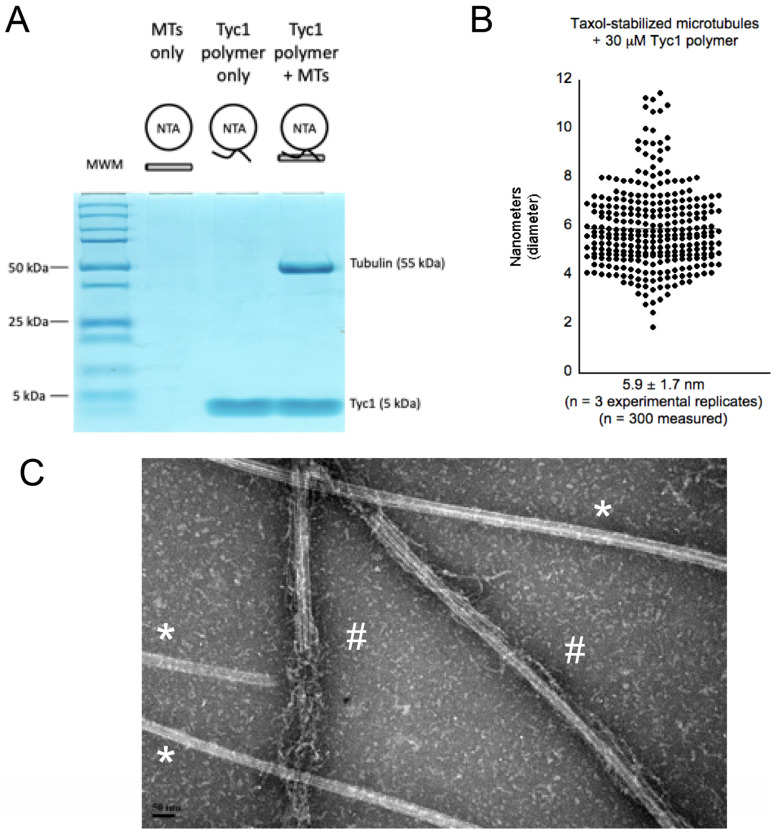
Tyc1p forms Taxol-stabilized microtubule-associated polymers. For simplicity and consistency, Tyc1p is denoted as “Tyc1” in the diagram. (**A**) An NTA-bead pull-down assay suggests that 50 μM Tyc1p has the ability to associate with Taxol-stabilized microtubules. (**B**,**C**) 30 μM Tyc1p alone forms polymers with a diameter of 5.9 ± 1.7 nm. Tyc1p polymers can extend off the ends of microtubules in a “comet-tail” shape (polymers marked by an # symbol) where the image also shows adjacent microtubules that do not have any Tyc1p polymers associated with them (marked with *). The scale bar in (**C**) is 50 nm.

**Figure 4:**
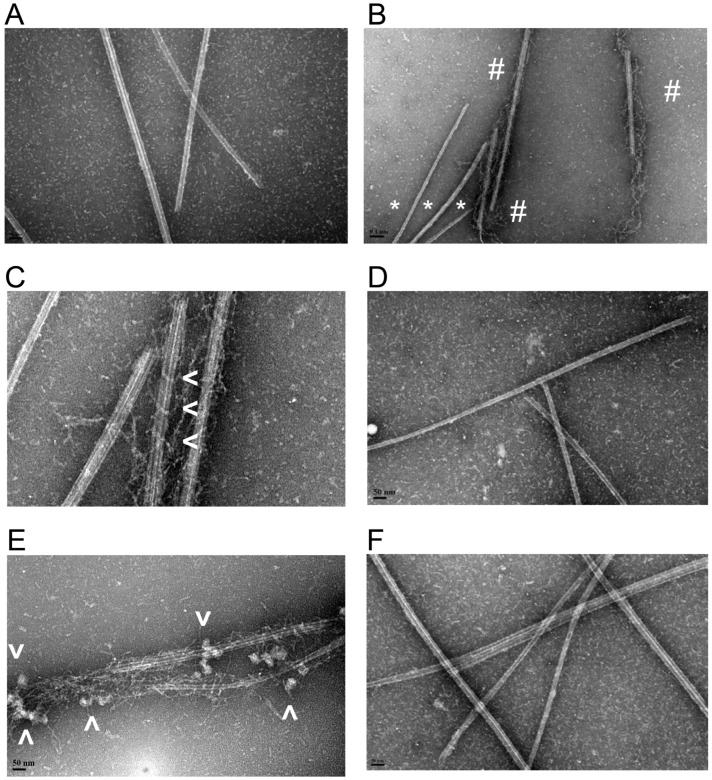
Tyc1p forms Taxol-stabilized microtubule-associated polymers in the presence of Mad2p-6xhis and a Cdc20p-derived Mad2-binding motif peptide [Mad2p-6xhis + DQ36] + Tyc1p in a manner that depends upon two highly conserved domains that share sequence homology with p31^comet^ in the p31^coment^ microtubule binding domain as revealed by [Mad2p-6xhis + DQ36] + Tyc1p-mut1 and [Mad2p-6xhis + DQ36] + Tyc1p-mut2 analyses. (**A**) The Mad2p-6xhis-DQ36 complex alone does not form any polymers. The scale bar is 50 nm. (**B**) [Mad2p-6xhis + DQ36] + Tyc1p form extended polymers associated with microtubules (marked with the # symbol) while in the presence of adjacent microtubules without any polymers associated with them (marked with an *). The scale bar is 100 nm in the image at 80,000×. (**C**) A higher magnification (150,000×) of the central region shown in (**B**) to highlight the nature of the polymers which sometimes appear to twist around the long axis of the microtubule (marked with <). (**D**,**E**) Mixtures of [Mad2p-6xhis + DQ36] + Tyc1p-mut1 very rarely formed polymers, and only in a few cases were polymers visible, but they were always in association of nonspecific globular structures (marked with arrows in (**E**) as an example), raising the concern they may be artifacts. (**F**) [Mad2p-6xhis + DQ36] + Tyc1p-mut2 was never observed to promote the formation of polymers. The scale bar is 50 nm.

Because p31^comet^ has been observed to co-localize with microtubule plus-ends in vivo and a Taxol-stabilized microtubule binding domain has been defined in vitro in p31^comet^ that shares a region of homology (EDWFRPKLNYRVPSRGHKLTVTLS) with Tyc1p (DDWFSRKFSKAVHGNNHGTISLS), we decided to begin our investigation of Tyc1p polymers by exploring their potential interaction with Taxol-stabilized microtubules [7]. We first tested the ability of 50 μM Tyc1p to promote an association between Taxol-stabilized microtubules and NTA beads where a positive pull-down activity was observed prompting us to investigate this interaction by negative staining EM analyses (Figure 3). In the presence of Taxol-stabilized microtubules, long curvilinear braided strands of Tyc1p polymers with an average diameter of 5.9 nm ± 1.7 nm were observed that have the appearance of comet-shapes that were often extending off the end(s) of the microtubules for up to several micrometers (Figure 3C). Tyc1p is known to physically interact with Mad2p [5,11]. We, therefore, co-incubated Tyc1p with pure recombinant Mad2p-6xhis protein that had been associated with the Cdc20p-derived DQ36 Mad2-binding motif peptide of budding yeast [1]. Initially, this incubation method disrupted the ability of Mad2p-6xhis to interact with the co-incubated Mad2p-binding motif peptide DQ36 (see Figure S1). To work around this disruption, we devised a polymer-assembly protocol where the Mad2p-6xhis-DQ36 complex was isolated first using a spin column and then subsequently exposed to 30 μM Tyc1p (see Figure S2). This subsequent exposure to 30 μM Tyc1p suppresses the ability of the Mad2-DQ36 complex to transit through a second spin column, likely due to polymer formation as exposure to Tyc1p also caused a shift in the sedimentation of Mad2p-6xhis into a high-density portion of a sucrose gradient (Figure S3). The 7.3 μM [Mad2p-6xhis + DQ36] + 30 μM Tyc1p mixture was then utilized for polymer formation, and evidence was again observed for robust polymer formation by negative staining EM in the presence of Taxol-stabilized microtubules (Figure 4A–C).

To identify which Tyc1p amino acid motifs may contribute to polymer formation, we investigated the activities of two mutant versions of the protein, “Tyc1p-mut1” (MKVLAAAASRKFSKAVHGNNHGTISLSTLSYIRVHKLVK) and “Tyc1p-mut2” (MKVLDDWFSRKFSKAVHGNNHGAAAAATLSYIRVHKLVK), with the most highly conserved amino acids changed to alanines. We incubated pure Tyc1p, Tyc1p-mut1, or Tyc1p-mut2 (at 30 μM) with Taxol-stabilized microtubules under our standard established conditions and observed the proteins by negative staining. In contrast to Tyc1p, Tyc1p-mut1 very rarely formed polymers, but they were always associated with non-specific aggregates in the micrographs, raising concerns that these Tyc1p-mut1 polymers may be artifacts. Tyc1p-mut2 did not form any polymers at all in the presence of Taxol-stabilized microtubules (Figure 4D–F). We have also observed that Tyc1p-mut1 and Tyc1p-mut2 cannot promote the disassociation of Mad2p-6xhis and DQ36, and that they can only partially suppress the ability of the Mad2p-6xhis -DQ36 complex from transiting through a second spin column (Figures S1 and S4). In combination, these observations suggest that the amino acid residues between Tyc1p and p31^comet^ make a contribution to polymer formation and likely towards the ability of Tyc1p to interact with microtubules.

**Figure 5:**
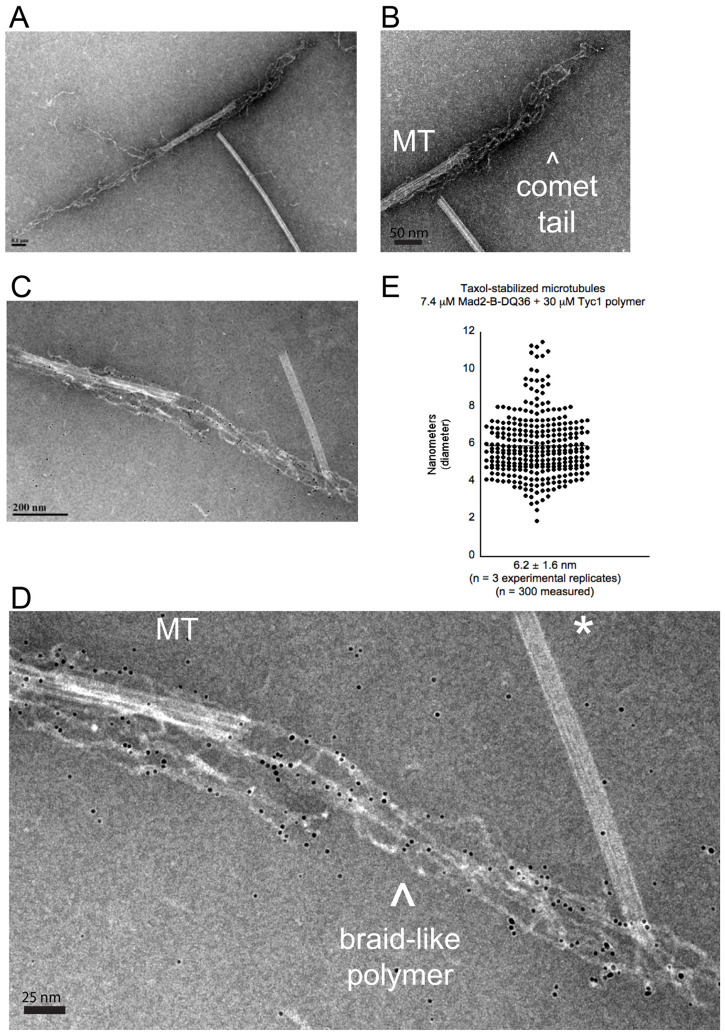
Ni-NTA 5 nm gold particles label the [Mad2p-6xhis + DQ36] + Tyc1p polymers. (**A**,**B**) The initial images at 80,000× and 15,000× are samples that were not exposed to the nano-gold particles as a control and highlight the comet-tail nature of the polymers as denoted with the arrow. The scale bar in (**A**) is 0.1 μm. (**C**–**E**) Two different magnifications of the same image are shown to indicate the overall labeling pattern of a microtubule (MT) or an adjacent unlabeled microtubule (denoted with an *). The 5 nm gold particles were associated with polymers that displayed an average diameter of 6.2 ± 1.6 nm. The braid-like nature of the individual polymer strands is also evident in the comet-tail of the polymers as denoted with an arrow.

**Figure 6:**
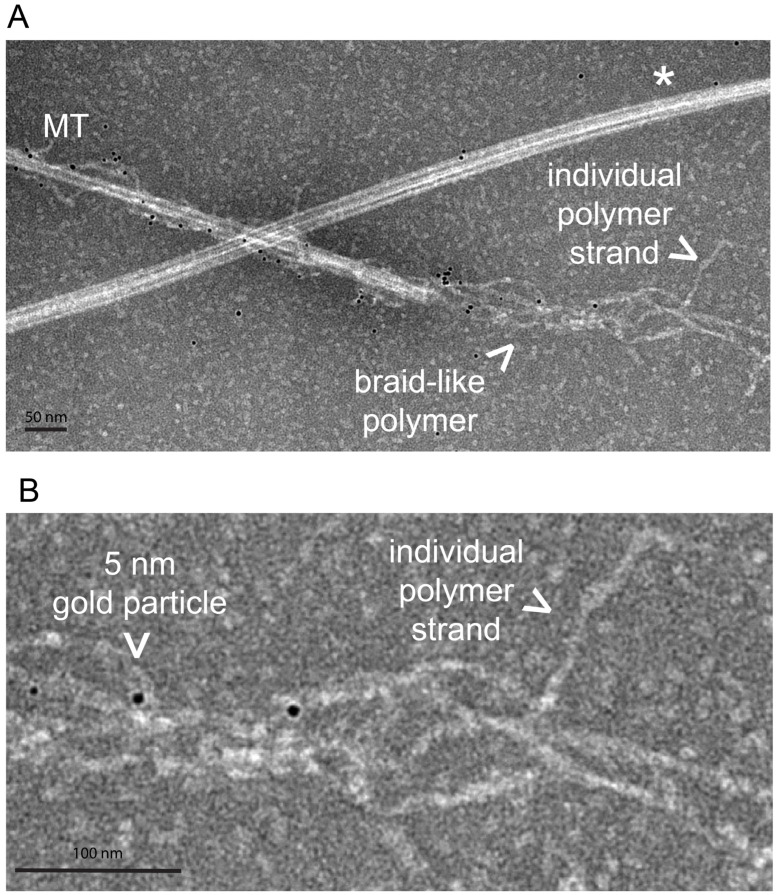
Ni-NTA 5 nm gold particles label the [Mad2p-6xhis + DQ36] + Tyc1p polymers. (**A**,**B**) To ensure the specificity of the nano-gold particle labeling, two different protocols were employed. In the results of the second protocol displayed here, the nano-gold particles were used at 1/100th of the original level, but the labeling incubation time was increased up to 8 min, yielding a more specific labeling outcome. A microtubule without any associated polymer is also visible as marked with the asterisk *. The braided nature of the polymers extending off the ends of the microtubule is apparent as marked with the arrows.

We next probed [Mad2p-6xhis + DQ36] + Tyc1p polymers with Ni^2+^-NTA-nanogold particles (5 nm). We observed an enrichment of labeling of comet-shaped polymers associated with the Taxol-stabilized microtubules (Figure 5 and Figure 6). Microtubules that do not have any associated polymer structures did not display any enrichment of labeling.

### 2.2. Tyc1p Polymers Suppress the Depolymerization of Taxol-Stabilized Microtubules Induced by Ice-Cold CaCl_2_

**Figure 7:**
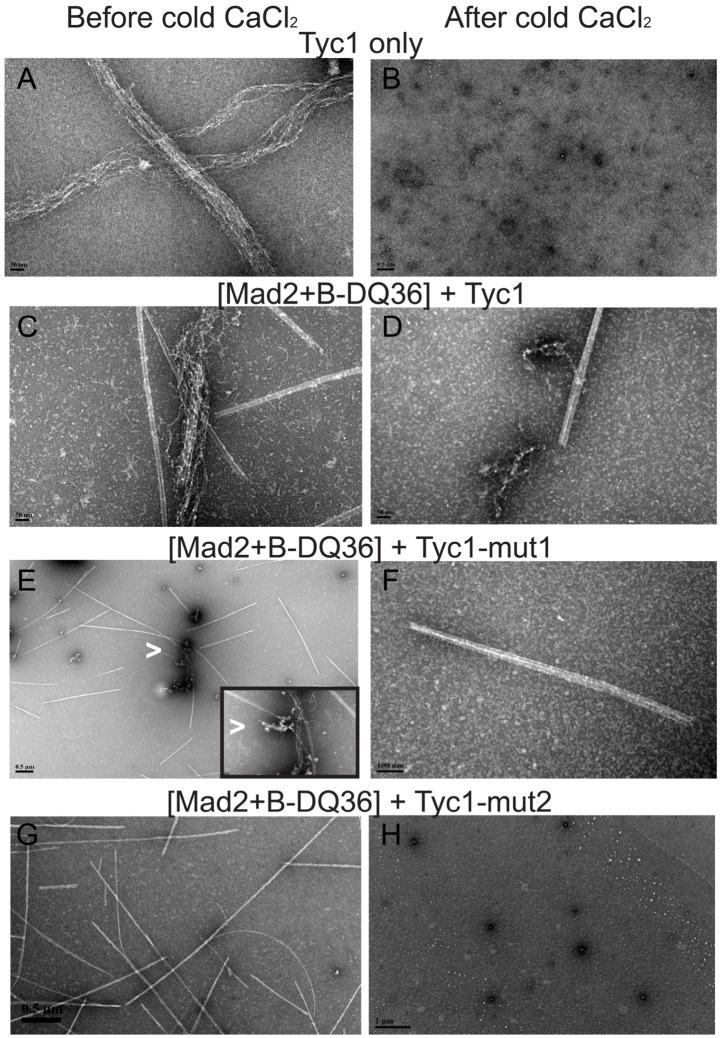
[Mad2p-6xhis + DQ36] + Tyc1p polymers, but not the Tyc1p polymer alone, can suppress Taxol-stabilized microtubule depolymerization induced by exposure to ice-cold CaCl_2_. Micrographs of samples after 1 h of ice-cold CaCl_2_ treatment are shown here. (**A**,**B**) Tyc1p polymers alone cannot suppress microtubule depolymerization induced by ice-cold CaCl_2_. The scale bar in (**A**) is 50 nm and in (**B**) is 500 nm. (**C**,**D**) [Mad2p-6xhis + DQ36] + Tyc1p polymers can suppress microtubule depolymerization induced by ice-cold CaCl_2_. The scale bars in both (**C**,**D**) are 50 nm. (**E**,**F**) [Mad2p-6xhis + DQ36] + Tyc1p-mut1 polymers (see footnote in Table 1) can only slightly suppress microtubule depolymerization induced by ice-cold CaCl_2_. Before ice-cold CaCl_2_ treatment, polymers were only observed in association with non-specific globular structures as marked with the arrow. The scale bar in (**E**) is 500 nm and in (**F**) is 100 nm. (**G**,**H**) [Mad2p-6xhis + DQ36] + Tyc1p-mut2 did not suppress microtubule depolymerization induced by ice-cold CaCl_2_. The scale bar in (**G**) is 500 nm and in (**H**) is 1000 nm.

Having observed Tyc1p only or [Mad2p-6xhis + DQ36] + Tyc1p polymers in association with Taxol-stabilized microtubules, we next investigated a possible function for this association, namely the suppression of microtubule depolymerization. Towards this end, the Taxol-stabilized microtubule-containing mixtures were exposed to 4 mM CaCl_2_ for 1 h on ice to induce depolymerization in the absence or presence of Tyc1p polymers or [Mad2p-6xhis + DQ36] + Tyc1p polymers and then analyzed before and after treatment by negative staining EM (Table 1). In the absence of added Tyc1p, no microtubules were observed after ice-cold CaCl_2_ treatment. For example, before ice-cold CaCl_2_ treatment, we observed 90 microtubules present in 3 micrographs, but 0 microtubules present after treatment. This assay should serve as a severe challenge to test if any polymer has the capacity to suppress microtubule depolymerization, as the expectation is that 100% of the microtubules should depolymerize after the ice-cold CaCl_2_ treatment. When only [Mad2p-6xhis + DQ36] was added to the samples, no microtubules were observed after ice-cold CaCl_2_ treatment. For example, before ice-cold CaCl_2_ treatment, we observed 93 microtubules present in 3 micrographs, but 0 microtubules present after treatment. In the presence of Tyc1p before ice-cold CaCl_2_ treatment, 7 micrographs containing 13 images of microtubules associated with Tyc1p polymers were observed demonstrating that the polymerization reaction was successful. After ice-cold CaCl_2_ treatment, no microtubules were observed in the micrographs (Table 1) (Figure 7A,B). However, by contrast, short microtubules were still observable in the presence of the 7.3 μM [Mad2p-6xhis + DQ36] + 30 μM Tyc1p polymers. Before ice-cold CaCl_2_ treatment, 26 micrographs containing 50 microtubules associated with polymers were observed demonstrating that the polymerization protocol worked, and 12 micrographs containing 20 microtubules were observed after ice-cold CaCl_2_ treatment (Table 1) (Figure 7C,D). We further investigated if [Mad2p-6xhis + DQ36] + Tyc1p-mut1 or [Mad2p-6xhis + DQ36] + Tyc1p-mut2 polymers had the ability to suppress Taxol-stabilized microtubule depolymerization induced by ice-cold CaCl_2_ treatment. The 7.3 μM [Mad2p-6xhis + DQ36] + 30 μM Tyc1p-mut1 display a slight ability to suppress Taxol-stabilized microtubule depolymerization, as a total of 4 microtubules in 3 micrograph images were observed remaining after treatment with one shown here (Table 1) (Figure 7E,F). By contrast, 7.3 μM [Mad2p-6xhis + DQ36] + 30 μM Tyc1p-mut2 had no ability to suppress Taxol-stabilized microtubule depolymerization (Table 1) (Figure 7G,H).

## 3. Discussion

Tyc1p forms spontaneous short linear individual strand-like polymers at a concentration of about 30 μM. In the presence of Taxol-stabilized microtubules, these strands appear to assemble into polymers that wrap around the associated microtubules and in the braided form appear to extend off the end of microtubules as long comet-shaped polymers. These polymers appear to be able to extend many micrometers in length under the assembly conditions used here both in association with a microtubule and off each end. However, the length of individual polymer strands remains difficult to quantify. The polymers have the appearance of being assembled on a few of the microtubules in a sample, while not assembling on others. This may result from stochastic nucleation of polymer filament growth under our assembly conditions. The individual strand width is approximately 6 nm with and without the [Mad2-6xhis + DQ36] protein present during polymer assembly, a width that is similar in magnitude as actin filaments (microfilaments). Nano-gold labeling revealed Mad2-6xhis and/or Tyc1p is present in the polymers, but we cannot differentiate between these possibilities as both Mad2-6xhis and Tyc1p associate with the NTA functional group.

The ability to suppress Taxol-stabilized microtubule depolymerization resides in the conserved 10 amino acid HGTISLSTL motif of Tyc1p at the heart of Tyc1p-mut2 mutant protein, a motif that is homologous with p31^comet^ HKLTVTLS. This motif notably resides within the mapped Taxol-stabilized microtubule-binding domain in the C-terminus of p31^comet^ [7]. However, the mechanism by which Tyc1p suppresses Taxol-stabilized microtubule depolymerization remains to be determined. Our hypothesis is that Tyc1p polymers that wrap around the Taxol-stabilized microtubules may create a mild physical barrier against depolymerization which requires the microtubule proto-filaments to curl back away from the central axis of the microtubule long axis. Other in vivo interactors are also likely to play an important biological role in this context too, such as Abm1p and Bim1p/EB1.

**Figure 8:**
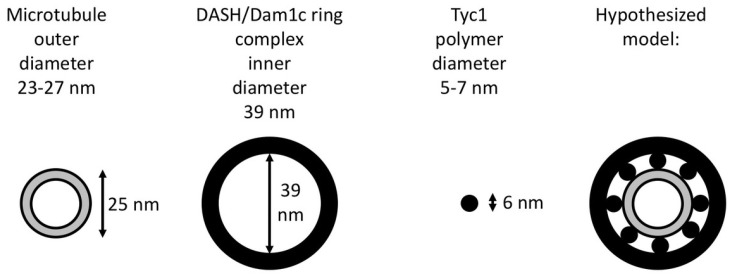
A model showing an end-on view for how Tyc1p-containing polymers may associate with a microtubule plus-end near a kinetochore. For simplicity and consistency, Tyc1p is denoted as “Tyc1” in the diagram. The model is based upon the measured outer diameter of microtubules (25 nm), the measured inner diameter of the DASH/Dam1c ring complex from fungi (39 nm) and the measured diameter of Tyc1p-containing polymers reported here (6 nm) [16,17].

Tyc1p has been reported to have physical interactions with Mad2p and Abm1p, and a functional interaction with Bim1p/EB1, all proteins that relate to aspects of microtubule function. Bim1p/EB1, a member of the highly conserved EB1 family of microtubule associated proteins, is a founding member of the plus-end tracking proteins (+TIPs) that localize to and ‘track’ the growing plus-ends of microtubules [14]. Bim1p/EB1 has numerous genetic and physical interactions in yeast including with Mad2p, components of the DASH/Dam1c ring complex, components of the kinetochore and with the Dynein/Dynactin motor protein complex, among many others [2,3,4]. The EB1 protein specifically localizes to the plus-ends of microtubules and has been co-localized with p31^comet^ in human cells [7]. Abm1p, erroneously labeled as BM1 in the original paper, was observed to be required for proper microtubule length in vivo, where *abm1* mutant cells displayed shorter microtubules [15]. Abm1p overexpression also increases chromosome loss rates and the *ABM1* gene has genetic interactions with gamma tubulin (*TUB4*), *DAM1* of the DASH/Dam1c ring complex at the kinetochore, and with *APC5*, a subunit of the APC/C [2,3,4,16,17]. Tyc1p working in concert in vivo with its functional partners such as Bim1p/EB1 at microtubule plus-ends and Abm1p, which are required to promote microtubule stability, may participate in promoting proper mitotic spindle microtubule mechanics, especially at microtubule plus-ends near kinetochores (Figure 8).

**Figure 9:**
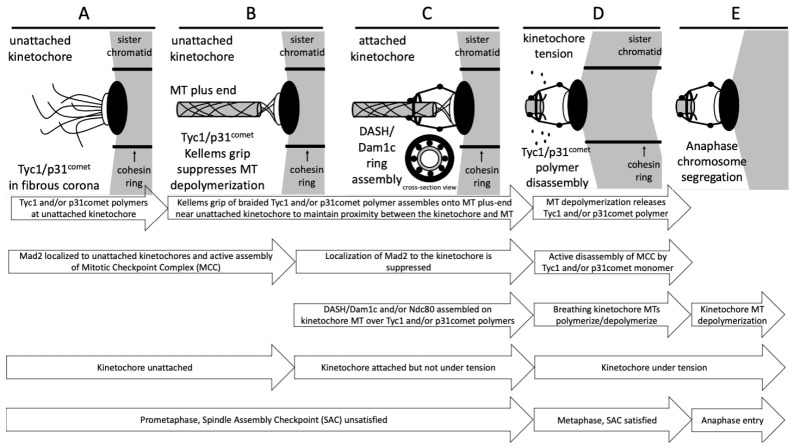
A set of hypotheses to test focused on Tyc1p and/or p31^comet^ functioning as polymers in the context of mitotic cell cycle progression. For orientation relative to detailed cell cycle progression events, below each diagram arrows indicate the timeline of mitotic events. For simplicity and consistency, Tyc1p is denoted as “Tyc1” in the diagram. (**A**) Prophase kinetochore with outer fibrous corona that may contain Tyc1p or p31^comet^. (**B**) An unattached prophase kinetochore associated with Tyc1p or p31^comet^ polymers induced to form a braid-like structure by a near-by microtubule plus-end where the Tyc1p or p31^comet^ polymer may function as a Kellems grip. (**C**) Prophase kinetochores become attached after the assembly of the DASH/Dam1c ring complex, but the kinetochore is not yet under tension. (**D**) In metaphase, microtubule depolymerization and ATP-dependent motor protein activity during ‘chromosome breathing’ generates kinetochore tension and promotes the disassembly of Tyc1p or p31^comet^ polymer into the soluble form that may be free to diffuse and thus promote the removal of Cdc20p from Mad2p leading to the spindle-assembly checkpoint being “satisfied”. (**E**) Anaphase entry and the advancement of the cell cycle after removal of sister-chromatid cohesion. It should be noted that DASH/Dam1c kinetochore rings have only been reported in fungi.

A puzzle remains: why would a potential cell cycle regulatory factor like Tyc1p and/or potentially p31^comet^ connect to mitotic spindle microtubule mechanics in the form of a polymer? We hypothesize that assembling a cell cycle regulatory protein into a polymer may create a pool of temporarily physically trapped proteins that cannot freely diffuse. Upon polymer disassembly, which is induced by the development of tension at kinetochores through microtubule depolymerization in conjunction with motor enzyme activity, the released subunits could then be free to diffuse and execute their biochemical activity. Influenced in part by ideas proposed by Ide, et al., 2023, where they proposed that the disassembly of the outer kinetochore fibrous corona contributes to the spindle assembly checkpoint being “satisfied”, we speculate that this may be a way for cells to couple mitotic mechanics with regulation of mitotic cell cycle progression [18]. We have drawn out a series of illustrations to express this hypothesis (Figure 9). This is not, in a formal sense, a tension-sensing mechanism, but rather illustrates a mechanism by which the development of tension at kinetochores may convert Tyc1p/p31^comet^ from a suppressed polymer form into a freely diffusing form of subunits that promotes cell cycle progression into anaphase. The braided nature of the Tyc1p comet-shaped polymers we have observed here may be meaningful as they are reminiscent in organization to the braided structure of a Kellems grip, also known as a “towing sock” or, colloquially, as the “Chinese finger trap”. A Kellems grip is a mechanical gripping device for cylindrical objects such as cables, pipes, or fingers. The Kellems grip was designed with the feature that the harder it is pulled on (i.e., an increase in tension along the long axis of the grip), the tighter the grip develops around the internal cylindrical object via the Poisson effect through compression in the direction perpendicular to the long axis (Figure 9). This type of mechanical feature has been reported for the budding yeast kinetochore [19]. To our knowledge, it has not been determined if p31^comet^ can form polymers, especially when interacting with MAD2, and, in this way might potentially contribute mechanically to the stabilization of microtubules near the kinetochore acting as part of an outer corona Kellems grip. Many HORMA-domain proteins are known to form polymers, and MAD2 is a founding member of this family. It will be intriguing to investigate in the future if p31^comet^ interacting with MAD2 may have a polymer form that can play a mechanical role that connects directly with their established roles in regulating cell cycle progression at the metaphase–anaphase transition.

## 4. Materials and Methods

### 4.1. Proteins and Peptides

The recombinant Mad2-6xhis was expressed in *E. coli* and purified following the manufacturer’s protocol via nickel-NTA-affinity purification as previously described (Qiagen, Hilden, Germany) [1]. The Cdc20 Mad2-binding motif peptide “DQ36” (DMNKRILQYMPEPPKCSSLRQKSYIMKKRTHYSYQQ) was synthesized and purified to 99% purity (Kelowna, Taipei, Taiwan) and dissolved in QAH buffer (20 mM HEPES at pH 8.0, 100 mM NaCl, and 1 mM MgCl_2_). Tyc1 protein (MKVLDDWFSRKFSKAVHGNNHGTISLSTLSYIRVHKLVK), “Tyc1-mut1” mutant protein (MKVLAAAASRKFSKAVHGNNHGTISLSTLSYIRVHKLVK) and “Tyc1-mut2” mutant protein (MKVLDDWFSRKFSKAVHGNNHGAAAAATLSYIRVHKLVK) were synthesized and purified to 99% purity (Kelowna, Taipei, Taiwan) and dissolved in QAH buffer [1].

### 4.2. Tyc1p Polymerization Assay with Mad2-6xhis + DQ36 Mad2-Binding Motif Peptide Mixture

Mad2-6xhis (59.2 µM final) was incubated with the Cdc20 Mad2-binding motif peptide DQ36 (50 µM final) in a final volume of 30 µL with QAH buffer for 1 h at room temperature and gently pipetted every 15 min. In total, 10 µL of this mixture was removed and diluted with 40 µL QAH buffer and incubated for an additional 15 min at room temperature. Micro Bio-Spin P-6 Gel Columns (Bio-Rad, Hercules, CA, USA) were washed and pre-equilibrated with BRB80 buffer (80 mM PIPES, 1 mM EGTA, 1 mM MgCl_2_, pH 6.9) at room temperature according to the manufacturer’s protocol (Bio-Rad, Hercules, CA, USA). It is essential to remove the Mad2-6xhis elution buffet by this desalting step because the QIAGEN elution buffer prevents the formation of polymers. Only DQ36 peptide that is interacting with the Mad2-6xhis will elute from the column, as the molecular weight of DQ36 is 4.5 kDa and the spin column has a molecular weight cut-off of 6 kDa. A total of 50 µL of the Mad2-6xhis + DQ36 mixture was added to a BRB80 pre-equilibrated column and centrifuged for 4 min at 1000× *g* at room temperature. Tyc1 protein was diluted with QAH buffer to a final concentration of 1 mM to make a stock solution at room temperature and then added to the Mad2-6xhis + DQ36 mixture isolated from the eluant from the Micro Bio-spin P-6 Gel column typically to a final concentration of 7.3 µM [Mad2-6xhis + DQ36] + 30 µM Tyc1p in our standard assays, approximately a 1:1:4 ratio of Mad2-6xhis:DQ36:Tyc1p for polymer formation.

### 4.3. Negative Staining Electron Microscopy and Nano-Gold Labeling

Mad2-6xhis (59.2 µM final) was incubated with DQ36 (50 µM final) in a final volume of 30 µL with QAH buffer for 1 h at room temperature and gently pipetted every 15 min. A total of 10 µL of this mixture was removed and diluted with 40 µL QAH buffer and incubated for an additional 15 min at room temperature. Micro Bio-Spin P-6 Gel Columns were washed and pre-equilibrated with BRB80 buffer (80 mM PIPES, 1 mM EGTA, 1 mM MgCl_2_, pH 6.9) at room temperature according to the manufacturer’s protocol. In total, 50 µL of the Mad2-6xhis + DQ36 mixture was added to the BRB80 pre-equilibrated column and centrifuged for 4 min at 1000× *g* at room temperature. Taxol (Sigma-Aldrich, St. Louis, MO, USA) was added to the eluent at a final concentration of 100 μM. For viewing Mad2-6xhis only, samples were placed onto a glow-discharged Formvar/carbon, 300 mesh copper grid (Pentad Scientific, Taipei, Taiwan) and processed as described below. To study interactions with microtubules, a pre-formed Taxol-stabilized microtubule kit was used (5 mg/mL) (Cytoskeleton Inc., Denver, CO, USA) by thawing mixtures directly in a 37 °C water bath for 10 min. Taxol-stabilized microtubules (1.2 mg/mL final, about 12 µM protein, average length of microtubules at 2 μm) were mixed with Mad2-6xhis + DQ36 (7.3 µM final) and Tyc1 protein (30 µM final; about 4× the amount of the Mad2-6xhis + DQ36) to a final volume of 16.5 µL and incubated at room temperature for 30 min. A total of 3 µL of the sample was dropped onto a glow-discharged Formvar/carbon, 300 mesh copper grids, and incubated for 30 s at room temperature and then the liquid was wicked away with filter paper #1 (Whatman, Marlborough, MA, USA). For nano-gold labeling, grids were placed upside-down on a droplet (5 µL) of 5 nanometer Ni-NTA-Nano-gold (Nanoprobes, Taipei, Taiwan) that was diluted 50-fold in BRB80 + 100 µM Taxol buffer and was left to stand in a humidity chamber at room temperature for 8 min, or a 5 µL drop of BRB80 + 100 µM Taxol for unlabeled samples. Following the manufacturer’s protocol, the grid was washed upside-down, placing the grid on a new droplet of 10 µL with BRB80 + 100 µM Taxol containing 8 mM imidazole (Sigma-Aldrich, St. Louis, MO, USA). The sample stood for 1 min, and liquid was blotted away with filter paper. The grid was washed a second time upside-down on a droplet of 10 µL with BRB80 + 100 µM Taxol. The sample stood for 30 s, the liquid was blotted away with filter paper, and the grid was removed from the humidity chamber. In total, 3 µL of 2% phosphotungstic acid (Nanoprobes, Taipei, Taiwan) was added and the sample was incubated for 30 s at room temperature, where the liquid was blotted away with filter paper. The sample was dried for 30 min at room temperature. Samples could be stored overnight in a dehumidifier if necessary. Samples were visualized using a JEOL JEM 1230 transmission electron microscope (JEOL Ltd., Tokyo, Japan). Taxol-stabilized microtubule preparations alone contained microtubules but also large globular structures. We avoided and excluded these globular structures in our analyses.

### 4.4. Depolymerizing of Taxol-Stabilized Microtubules with CaCl_2_ on Ice

Samples were prepared containing Taxol-stabilized microtubules (Cytoskeleton Inc., Denver, CO, USA) and 7.3 µM [Mad2-6xhis + DQ36] + 30 µM Tyc1p assembled as described above. After polymer assembly in the presence of microtubules, CaCl_2_ was added to samples to a final concentration of 4 mM and incubated on ice for 1 h to promote Taxol-stabilized microtubule depolymerization. Samples were then prepared for negative staining EM as described above.

### 4.5. NTA-Bead Pull-Down Assay

Pre-formed Taxol-stabilized microtubule kit was used (5 mg/mL) (Cytoskeleton Inc., Denver, CO, USA) by thawing mixtures directly in a 37 °C water bath for 10 min. Taxol-stabilized microtubules (1.2 mg/mL final, about 12 µM protein, average length of microtubules at 2 μm) were mixed with QAH buffer supplemented with Taxol (100 μM) or with Tyc1 protein (152 µM final concentration). The Tyc1 protein-only control was supplemented with BRB80 + 100 μM Taxol. Samples were incubated for 20–30 min at room temperature. NTA his-tag purification Dynabeads (Thermo Fisher Scientific, Waltham, MA, USA) were employed according to the manufacturer’s protocol. NTA beads were prepared by washing 3 times in QAH buffer. After washing, beads were suspended in 33 µL of QAH buffer supplemented with 100 µM Taxol. A total of 16.5 µL of the sample mixtures above were added to the beads to yield a final concentration of 50 µM Tyc1p a level that promotes polymer formation but does not yield aggregates as observed by negative staining electron microscopy. Samples + beads were incubated for 10 min at room temperature, and then beads were washed 5 times with QAH buffer supplemented with 100 µM Taxol. Beads were centrifuged at 13,000 rpm (about 12,000× *g*) for 30 s at 4 °C in a table-top Eppendorf centrifuge and the supernatant was removed before the final magnetic bead isolation step. Beads were boiled directly in protein sample buffer and run out on a 4–20% gradient SDS-PAGE gel following the manufacturer’s protocol (Bio-East Technology Co., Taipei, Taiwan) and stained with Coomassie blue.

## Figures and Tables

**Table 1:** The number of microtubules or microtubules (MTs) + polymers observed in micrographs before (left) and after (right) exposure to ice-cold CaCl_2_.

Added Protein	Micrographs	MTs Only or MTs + Polymers Before Ice-Cold CaCl_2_	Micrographs	MTs Remaining After Ice-Cold CaCl_2_
none	3	90 MTs only	n/a	0
7.3 μM [Mad2p-6xhis + DQ36]	3	93 MTs only	n/a	0
30 μM Tyc1p	7	13 MTs + polymer	n/a	0
7.3 μM [Mad2p-6xhis + DQ36] + 30 μM Tyc1p	26	50 MTs + polymers	12	20
7.3 μM [Mad2p-6xhis + DQ36] + 30 μM Tyc1p-mut1 *	8	0 * MTs + polymers	3	4
7.3 μM [Mad2p-6xhis + DQ36] + 30 μM Tyc1p-mut2	6	0 MTs + polymers	n/a	0

* No Tyc1p-mut1 polymers were ever observed independent of an association with non-specific globular structures as shown in Figure 4E or Figure 7E (below). By our analysis criteria, where we excluded these structures, as stated in the methods section, none of the observed 7.3 μM [Mad2p-6xhis + DQ36] + 30 μM Tyc1p-mut1 qualified as a polymer + MT structures. However, 4 microtubules were observed in the absence of an associated globular structure after the treatment with ice-cold CaCl_2_, which we document here. In the table “n/a” indicates that during the viewing of the samples in the electron microscope no images were collected because no microtubules were present.

## Data Availability

The raw data supporting the conclusions of this article will be made available by the authors on request.

## Supplementary Materials

The following supporting information can be downloaded at: https://www.mdpi.com/article/10.3390/ijms27125436/s1.
