# Supplementary material for: Tiny Yeast Comet 1-Dependent Polymers Suppress Taxol-Stabilized Microtubule Depolymerization Induced by Ice-Cold CaCl2
Source: Int J Mol Sci. 2026 Jun 16;27(12):5436. doi: 10.3390/ijms27125436 (PMC13299185; doi:10.3390/ijms27125436)
Supplement: Supplementary file 1 [file ijms-27-05436-s001.zip › ijms-4198992-supplementary.pdf]

Supplementary Material for, "Tiny Yeast Comet 1-dependent polymers suppress Taxol-stabilized microtubule depolymerization induced by ice-cold $CaCl_2$" [IJMS-4198992], by Schuyler, Chen and Weng (2026).

A

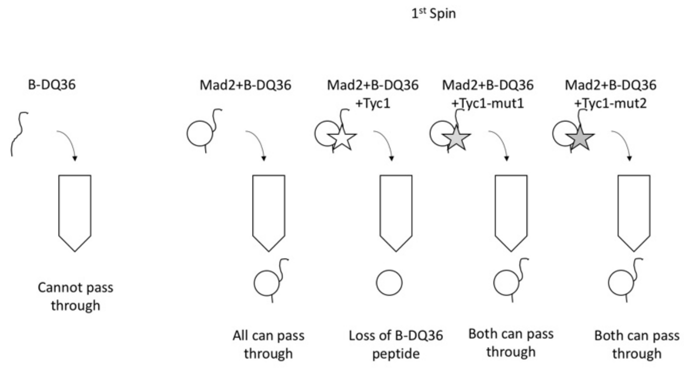

B

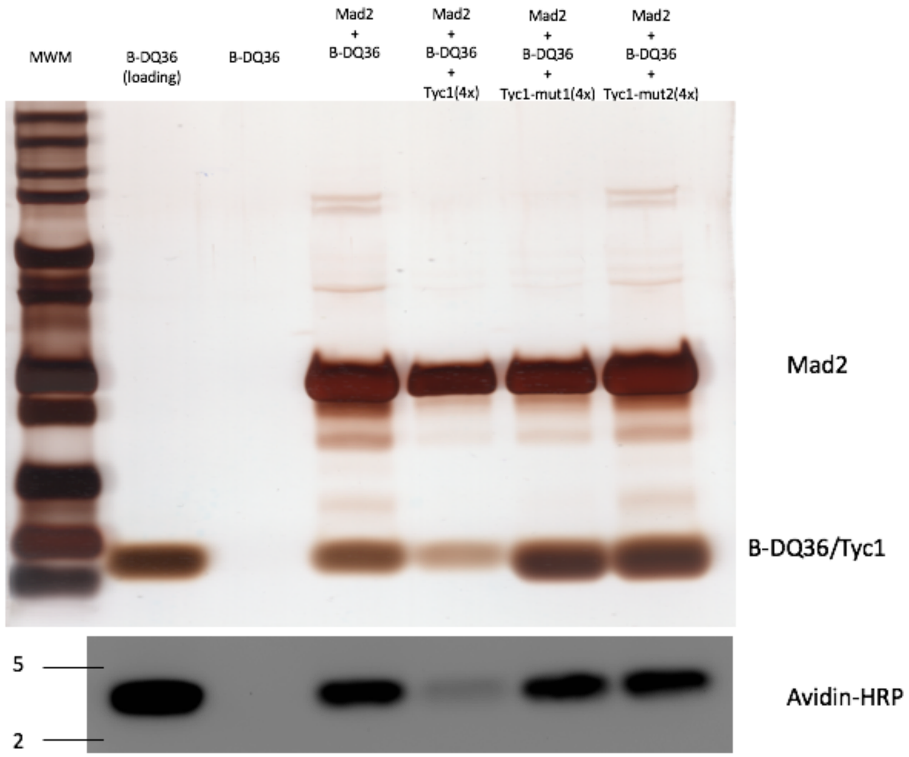

Figure S1. Wild type Tyc1 protein promoted the disassociation between Mad2p-6xhis and the Cdc20p Mad2-binding motif peptide biotinylated-DQ36 (B-DQ36) as observed by disrupting the ability of B-DQ36 to transit through the first spin column along with Mad2p-6xhis. For simplicity

within limited space, Mad2p-6xhis is denoted as “Mad2” and Tyc1p is denoted as “Tyc1” in the diagrams. **A)** A schematic diagram of the experimental design to test the results of exposing a mixture of Mad2p-6xhis and the B-DQ36 peptide to Tyc1 protein *before* the first spin column rather than *after* the first spin column. **B)** A silver-stained gel and Western blot revealed that upon exposure to Tyc1 protein *before* the first spin column, Tyc1p suppressed the association between Mad2p-6xhis and the Mad2p-binding motif peptide B-DQ36. In contrast, the Tyc1p-mut1 and Tyc1p-mut2 proteins failed to disrupt the interaction between Mad2p-6xhis and B-DQ36 peptide through the first spin column (n = 4).

A

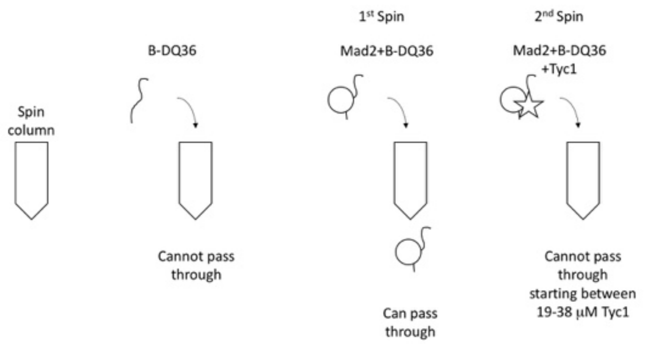

B

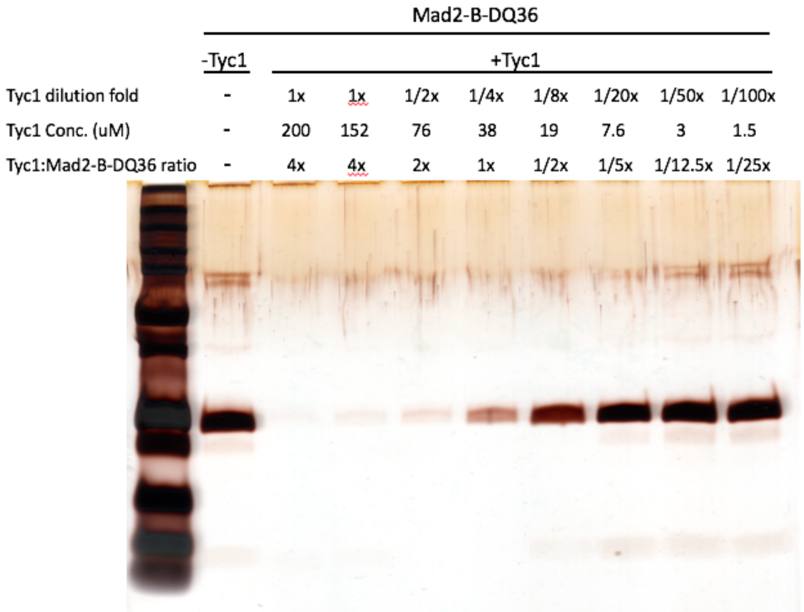

Figure S2. Tyc1p blocked the transit of the Mad2p-6xhis-B-DQ36 complex through the second spin column starting at a concentration of between 19 and 38 μM, which is the same molar range at which Tyc1p alone began to form the short polymer strands that were observed under negative staining electron microscopy (see **Figure 1 and 2**). For simplicity and consistency within limited space, Mad2p-6xhis is denoted as "Mad2" and Tyc1p is denoted as "Tyc1" in the diagrams. **A)** A schematic diagram the standard polymer assembly protocol. After the first spin column, the resultant Mad2p-6xhis-B-DQ36 complex is exposed to the Tyc1 protein which prevents the transit of Mad2p-6xhis-B-DQ36 in the second spin column. **B)** A silver-stained SDS-PAGE gel displaying a titration of Tyc1p to determine the level at which the Mad2p-6xhis-B-DQ36 was blocked from transiting the second spin column. On this gel, only the Mad2 protein

is visible, as the small molecular weight bands for the B-DQ36 peptide and Tyc1 protein were not visible at the leading edge of the gel front (n = 1).

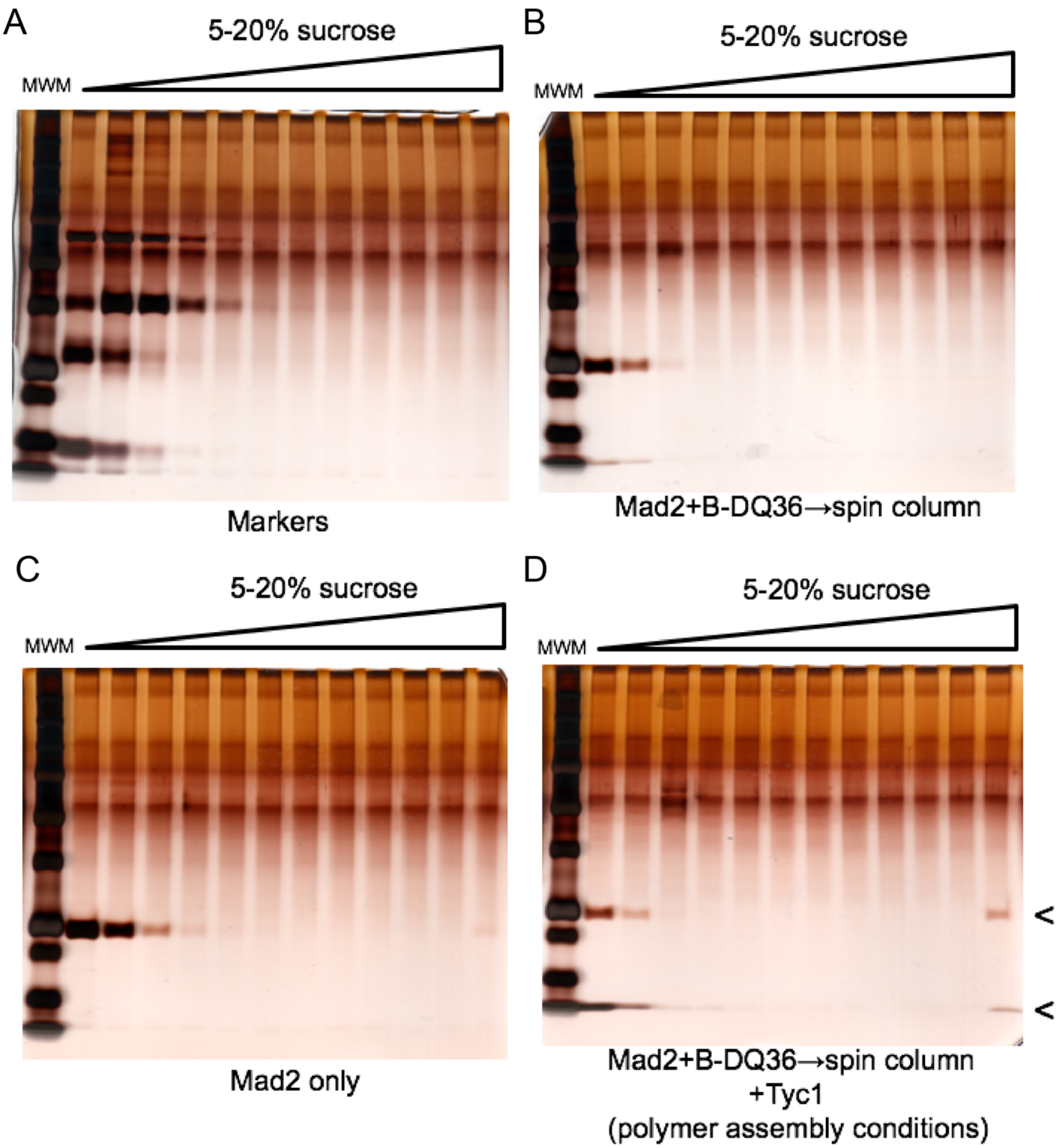

Figure S3. Silver-stained SDS-PAGE gels of sucrose density gradients (5-20%) displaying a shift in the sedimentation pattern of Mad2p-6xhis-B-DQ36 after exposure to Tyc1 protein. For simplicity and consistency within limited space, Mad2p-6xhis is denoted as “Mad2” and Tyc1p is denoted as “Tyc1” in the diagrams. **A)** A control sucrose gradient containing sedimentation protein markers including Catalase (monomer 60 kD, 11.3 S); Alcohol Dehydrogenase (150 kD, 7.7 S); Albumin (66 kD, 4.3 S), Carbonic anhydrase (29 kD, 2.8 S), and Cytochrome c (12.4 kD, 1.9 S). **B)** The Mad2p-6xhis-B-DQ36 (Cdc20p Mad2-binding motif peptide) complex after the

first spin column, which only contains B-DQ36 that is interacting with Mad2p-6xhis, remained in the low-density portion of the gradient in the first 1-2 fractions. **C)** Pure recombinant Mad2p-6xhis protein only. A small amount of Mad2p-6xhis was observed in the high-density sucrose fraction (far right) as bacterially-expressed recombinant Mad2 is known to form aggregates spontaneously at a low level. The small amount of Mad2p-6xhis aggregates are removed before our Tyc1-induced polymerization step in our standard polymer assembly assay after incubating Mad2p-6xhis with B-DQ36 and spinning them together through the first spin column, as no aggregated Mad2p-6xhis was observed in the sucrose gradient high-density fraction in the **Figure S2B** panel above. **D)** The Mad2p-6xhis-B-DQ36 complex in combination with Tyc1p. After passing through the first spin column, the Mad2p-6xhis-B-DQ36 was exposed to Tyc1 protein. A large portion of the Mad2p-6xhis (marked with an arrow "<" on the right) was shifted into the high-density sucrose fraction, which is consistent with polymer formation. A low molecular weight protein was also observed in the high density sucrose fraction that may indicate the presence of Tyc1 protein and/or the B-DQ36 peptide (marked with an arrow "<" on the right) (n=1).

A

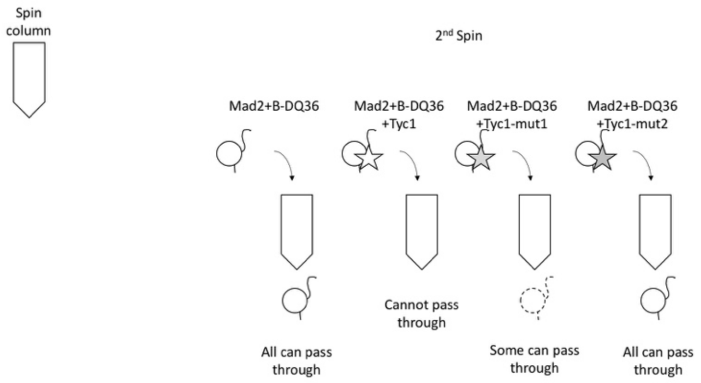

B

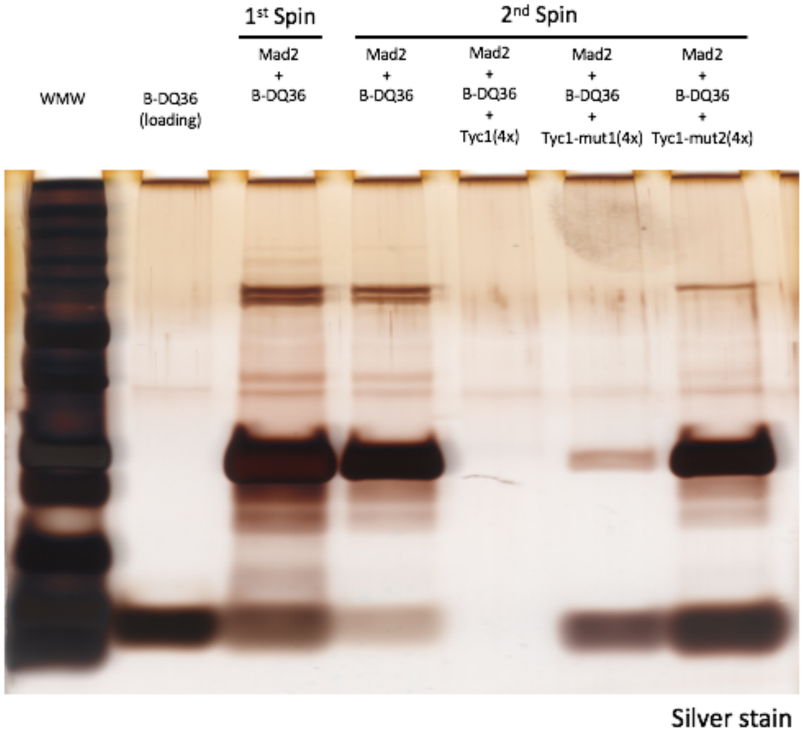

Figure S4. Tyc1 protein blocked the transit of the Mad2p-6xhis-B-DQ36 complex through a second spin column under our standard polymer assembly assay, while the mutant forms of Tyc1p did not. For simplicity and consistency within limited space, Mad2p-6xhis is denoted as "Mad2" and Tyc1p is denoted as "Tyc1" in the diagrams. **A)** A schematic diagram of our standard polymer assembly assay. **B)** An SDS-PAGE silver-stained gel revealing that in the presence of Tyc1 protein, none of the Mad2p-6xhis-B-DQ36 complex was observed to transit through a second spin column. In contrast, Tyc1p-mut1 could only partially block the transit of

the Mad2p-6xhis-B-DQ36 complex through the second spin column. In addition, Tyc1p-mut2 did not block the transit of the Mad2p-6xhis-B-DQ36 complex through the second spin column (n = 2).
